# Reduction of hospital-acquired MRSA as a result of increased use of hydoalcoholic handrub solution: a 7 year follow-up

**DOI:** 10.1186/1753-6561-5-S6-O87

**Published:** 2011-06-29

**Authors:** B Heyneman, E Nulens, A Lenez, S Pinxten, B Gordts, H Sax, D Pittet, Infection control team AZ Sint-Jan Brugge-Oostende

**Affiliations:** 1 IC team, AZ Sint-Jan Brugge-Oostende, Brugge, Belgium; 2 IC team, ZNA, Antwerpen, Belgium; 3 IC team, University Hospitals, Geneva, Switzerland

## Introduction / objectives

In the Sint Jan General Hospital, a 900 bed public and teaching hospital, incidence of hospital acquired MRSA (HA-MRSA) increased till 2003 to 5.5/1.000 admissions (adm). In 2004 VigiGerme®, a strategy to fight MRSA by reducing transmission was implemented in collaboration with the University Hospitals of Geneva, in order to reduce the HA-MRSA.

## Methods

According to standard dosages used and the quantity of Hydroalcoholic Handrub Solution (HAHS) consumed, the theoretical number of handrub actions could be calculated as an indicator for the hand hygiene (HH) compliance in hospital wards by registering the consumption of HAHS related to patient days. Besides, a bed-side measurement of compliance with hand hygiene was performed, indicating the number of opportunities for HH. Data registration and statistical analysis was performed with MS-Excel^®^ and SPSS^®^. Feedback was given per ward every 6 months. Day by day follow-up of MRSA attack-rate was performed by the infection control team and feedback and education was given per ward on regular base.

## Results

Implementation of the IC program resulted in significant increase of the use of HAHS by 81% (p<0.001). HA-MRSA significantly decreased from 5,5/1.000 adm in 2003 to 0,76/1.000 adm in 2010. Hospital-wide analysis indicates a clear correlation between HAHS consumption and decrease of HA-MRSA. Pearson correlation is statistically significant (-.839 p = .018). This suggests that increased use of HAHS is associated with lower HA-MRSA.

## Conclusion

During this hospital wide IC program, the significant increased use of HAHS correlated well with the decrease of HA-MRSA. These results indicate that consumption of HAHS is a good indicator for the decrease of HA-MRSA.

## Disclosure of interest

None declared.

